# Supplementary material for: Structural basis for the activation of a compact CRISPR-Cas13 nuclease
Source: Nat Commun. 2023 Sep 20;14:5845. doi: 10.1038/s41467-023-41501-5 (PMC10511502; doi:10.1038/s41467-023-41501-5)
Supplement: Supplementary file 1 — Supplementary Information [file 41467_2023_41501_MOESM1_ESM.pdf]

Supplementary Information

# Structural basis for the activation of a compact CRISPR-Cas13 nuclease

Xiangyu Deng[1,5], Emmanuel Osikpa[2,5], Jie Yang[2], Seye J. Oladeji[1], Jamie Smith[1], Xue Gao[2,3,4]*, Yang Gao[1]*

*Corresponding to yg60@rice.edu (Y.G.), xue.gao@rice.edu (X.G.)

This PDF file contains:

Supplementary Fig. 1-11

Supplementary Table 1-4

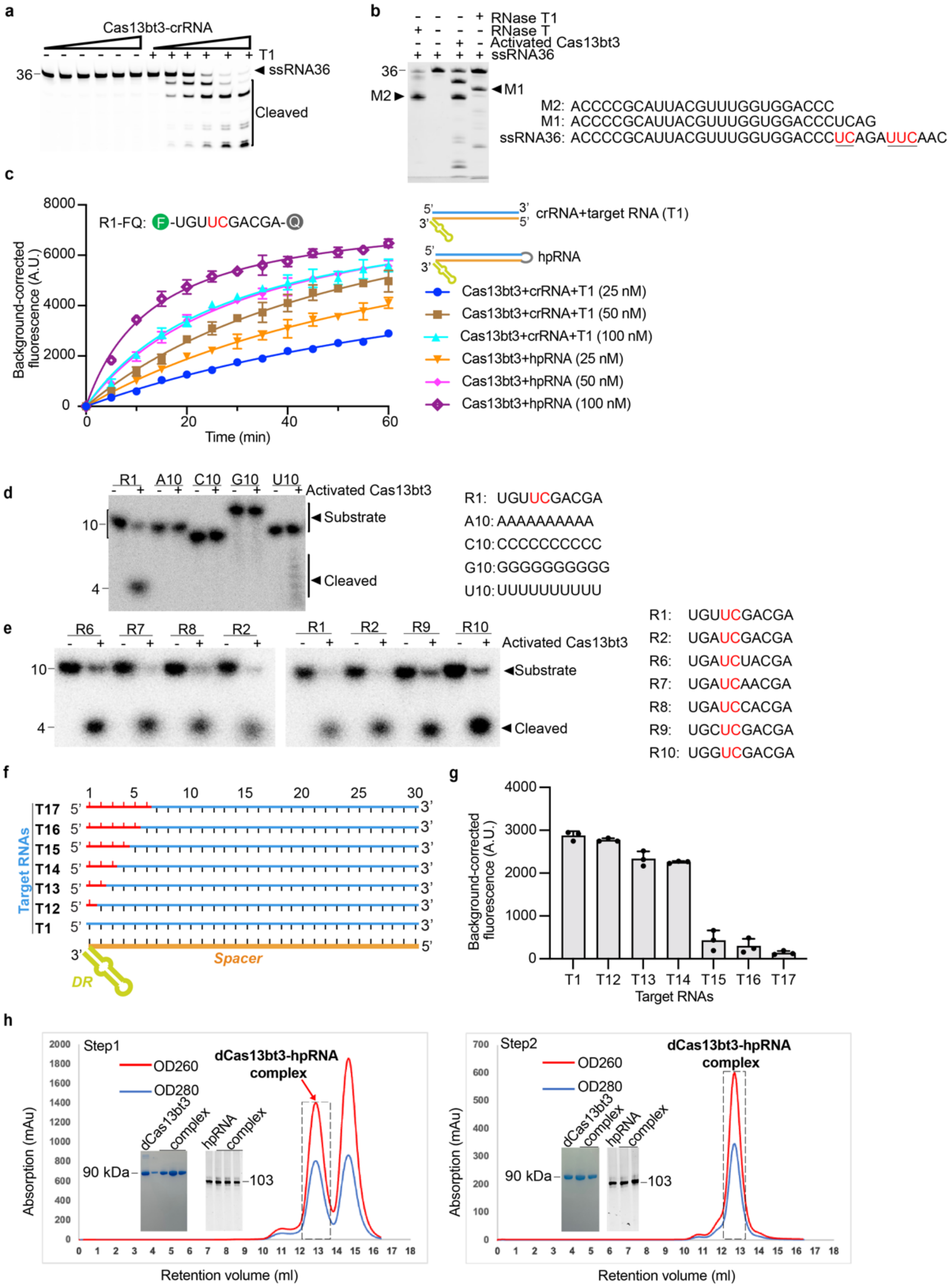


**Supplementary Fig. 1 Biochemical characterization of Cas13bt3 cleavage and purification of dCas13bt3-hpRNA complex.**

**a-b** Mapping the cleavage sites on ssRNA36. A representative gel image of ssRNA36 cleaved by 12.5-200 nM activated Cas13bt3 is shown in (**a**), and RNA size controls produced by RnaseT1 (M1) and RnaseT (M2) are in (**b**). The RNaseT1 specifically cleaves ssRNA36 at the G site to produce M1, whereas the RNaseT is a 3'-to-5' exonuclease that will stop at poly C motifs to produce M2. The possible cleavage sites on ssRNA 36 by Cas13bt3 according to the size comparison are indicated and colored in red. **c** Collateral activity of Cas13bt3 activated by crRNA-target RNA or hpRNA **d** Collateral activity of Cas13bt3 to ssRNA with poly-A10, poly-C10, poly-

G10, and poly-U10, R1 was used as a positive control. **e** Collateral activity of Cas13bt3 to ssRNA with UC cleavage site and various 5' or the 3' adjacent nucleotides. **f** Target RNAs designed for testing Cas13bt3 collateral activity with target RNAs 5' mismatches. The mismatched RNA segment is colored in red. **g** Collateral cleavage activities of Cas13bt3 with substrates in (**f**). (**c**, **g**) R1-FQ was used as reporter RNA. Data are present as means ± SDs (n=3). A.U. relative fluorescence intensity in arbitrary units. **h** The two-step purification of Cas13bt3-hpRNA complex for cryo-EM sample preparation (see methods for details). The purified complex indicated by bars in the chromatograms was checked with SDS-PAGE gel with protein control and Urea-PAGE gel with RNA control. Protein size (in kDa) and RNA size (in nt) are indicated. (**a**, **b**, **d**, **e**) RNA sizes (in nt) are indicated. At least three times each experiment was repeated independently with similar results. Source data are provided as Source Data File.

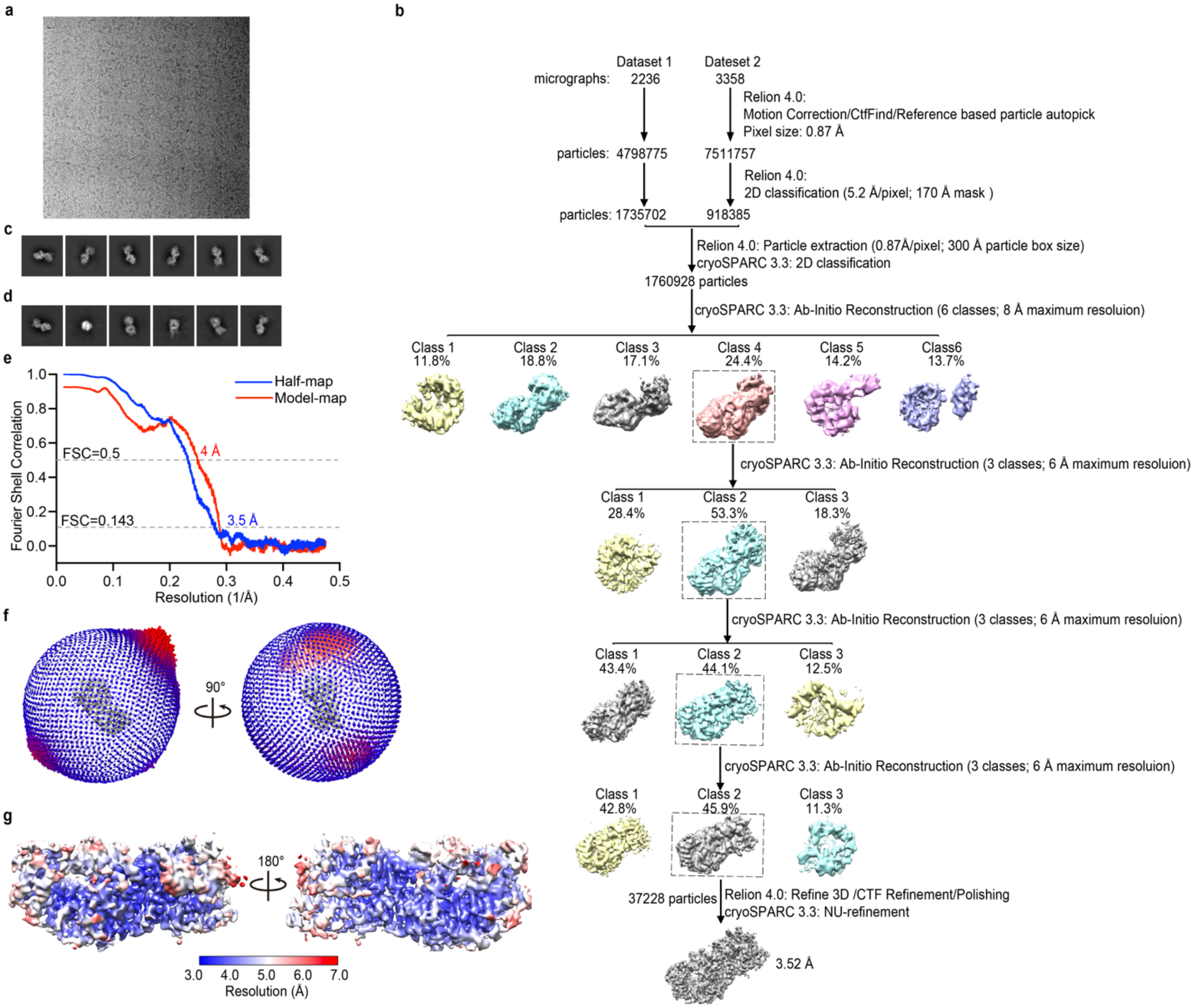


**Supplementary Fig. 2 Cryo-EM reconstruction of Cas13bt3-hpRNA complex.**
**a** representative cryo-EM micrograph. **b** Overview of image processing and refinement strategy. **c** Representative 2D class averages of Dataset 1 from QuantiFoil grid. **d** Representative 2D class averages of Dataset 2 from UltraAu grid. **e** Gold-standard FSC curves between the two half maps with indicated resolution at FSC = 0.143 are in blue. FSC curves between the refined model and the cryo-EM map with indicated resolution at FSC = 0.5 are in red. **f** The angular distribution of particles used in the final 3D reconstruction. **g** Local resolution estimation of the cryo-EM density map of Cas13bt3-hpRNA complex.

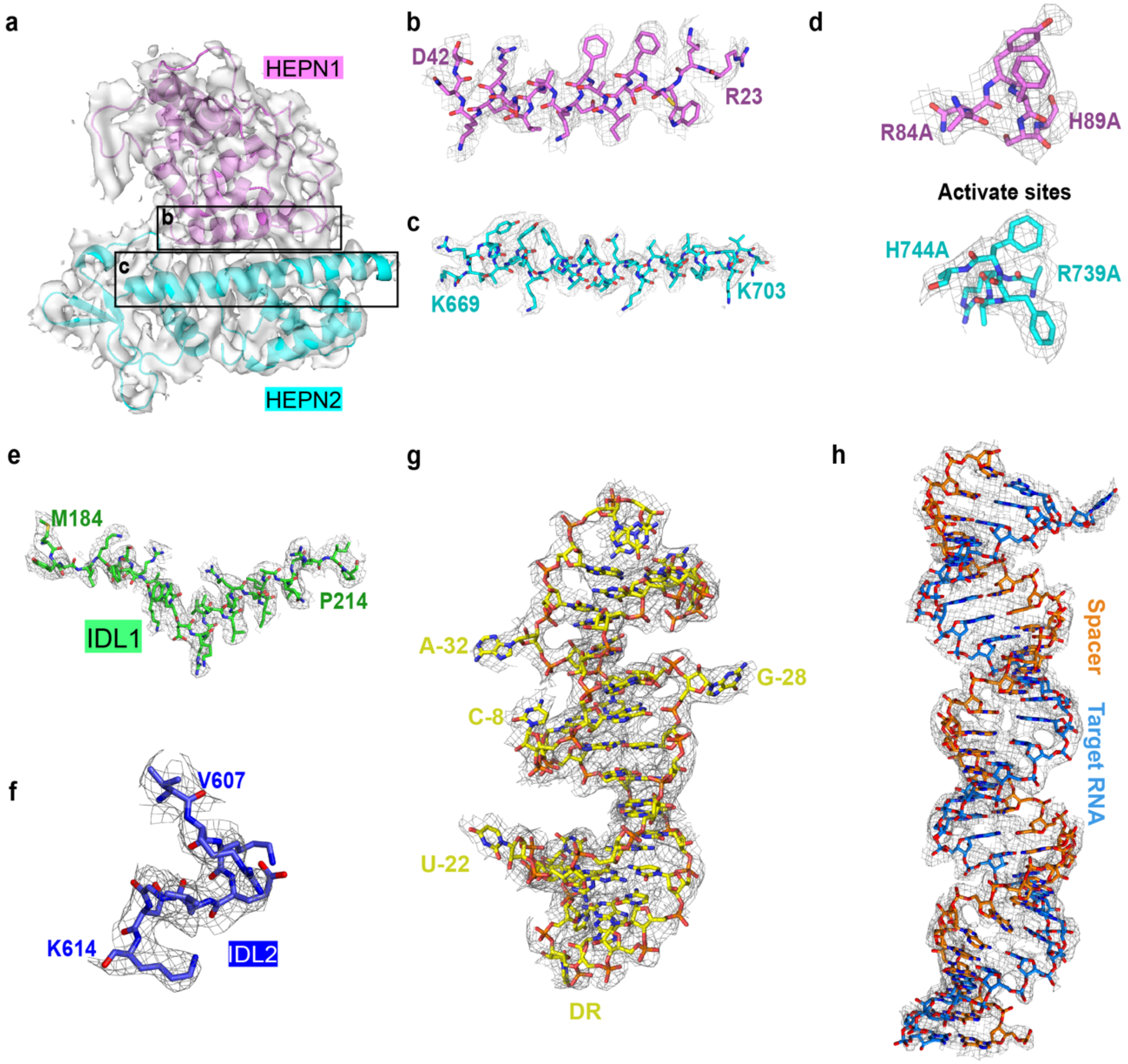


**Supplementary Fig. 3 Density map to the structure models of Cas13bt3-hpRNA complex.** **a** Cryo-EM density maps of the HEPN1 and HEPN2 domains in Cas13bt3$^{Act}$. **b-c.** Zoom in view of the local density map in (**a**). **d-h.** Cryo-EM density maps of activate sites (the RXXXXH motif) on HEPN domains, IDL1, IDL2, DR, and spacer-target RNA duplex in Cas13bt3$^{Act}$. Color codes are defined as in Fig. 1c and 1d, respectively.

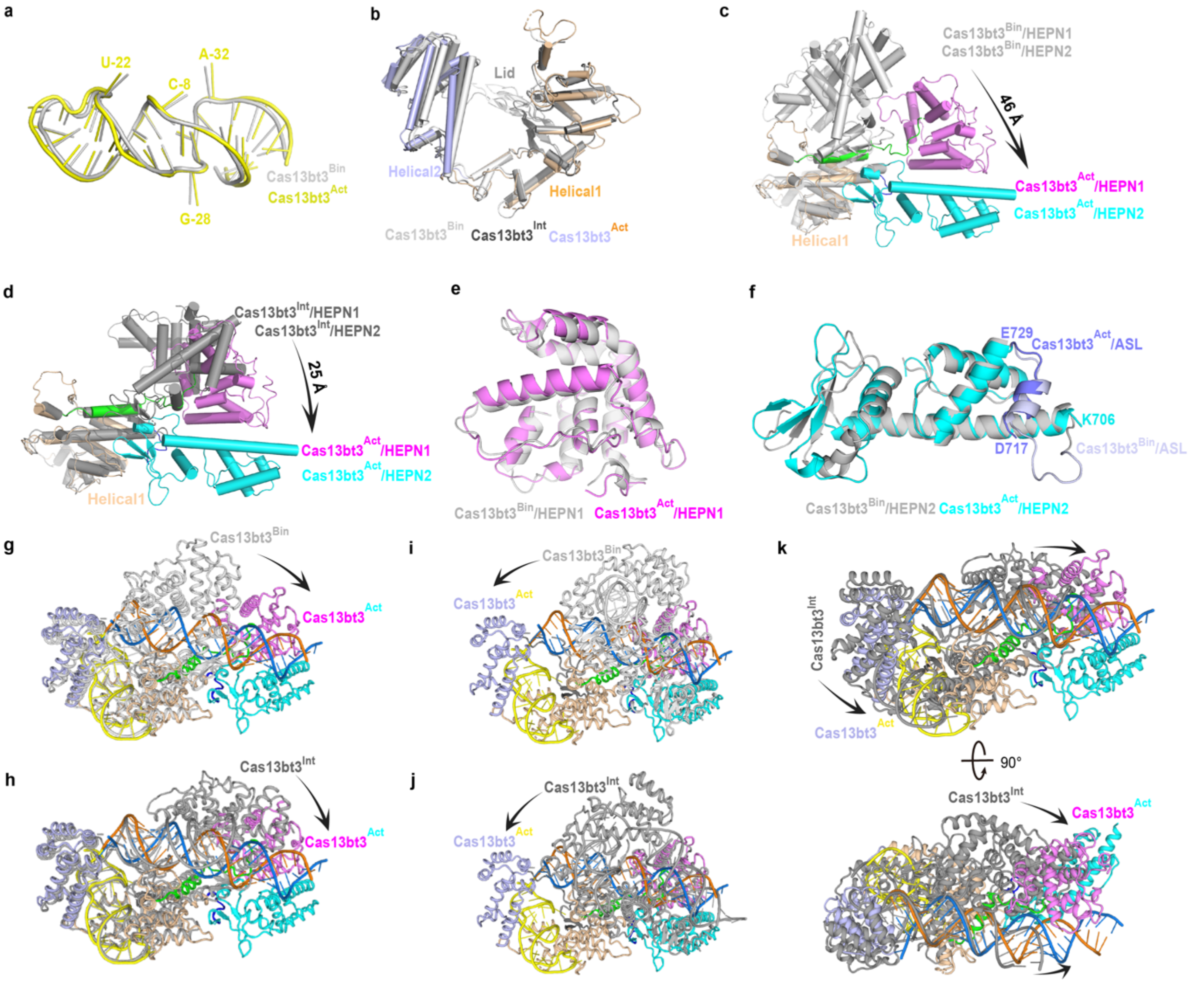


**Supplementary Fig. 4 Structural comparisons of Cas13bt3$^{Bin}$, Cas13bt3$^{Int}$ and Cas13bt3$^{Act}$.** **a** Structural alignment of DR in Cas13bt3$^{Bin}$ (grey) and Cas13bt3$^{Act}$ (yellow). **b** Structural alignment of REC lobe in Cas13bt3$^{Bin}$ (grey), Cas13bt3$^{Int}$ (black), and Cas13bt3$^{Act}$ (colored). **c-d** The conformational change of HEPN domains in Cas13bt3$^{Act}$ (colored), compare with Cas13bt3$^{Bin}$ (grey) (**c**) and Cas13bt3$^{Int}$ (black) (**d**), the distance of the NUC lobe's mass center movement was indicated. **e-f** Structural alignment of HEPN1 (**e**) and HEPN2 (**f**) in Cas13bt3$^{Bin}$ (grey) and Cas13bt3$^{Act}$ (colored). **g-k** Overall structural comparison of Cas13bt3$^{Bin}$ (grey), Cas13bt3$^{Int}$ (black) with Cas13bt3$^{Act}$ (colored) by fixing the REC lobe (**g-h**) and NUC lobe (**i-j**), or duplex RNA (**k**). The movements of the domains and RNAs were indicated.

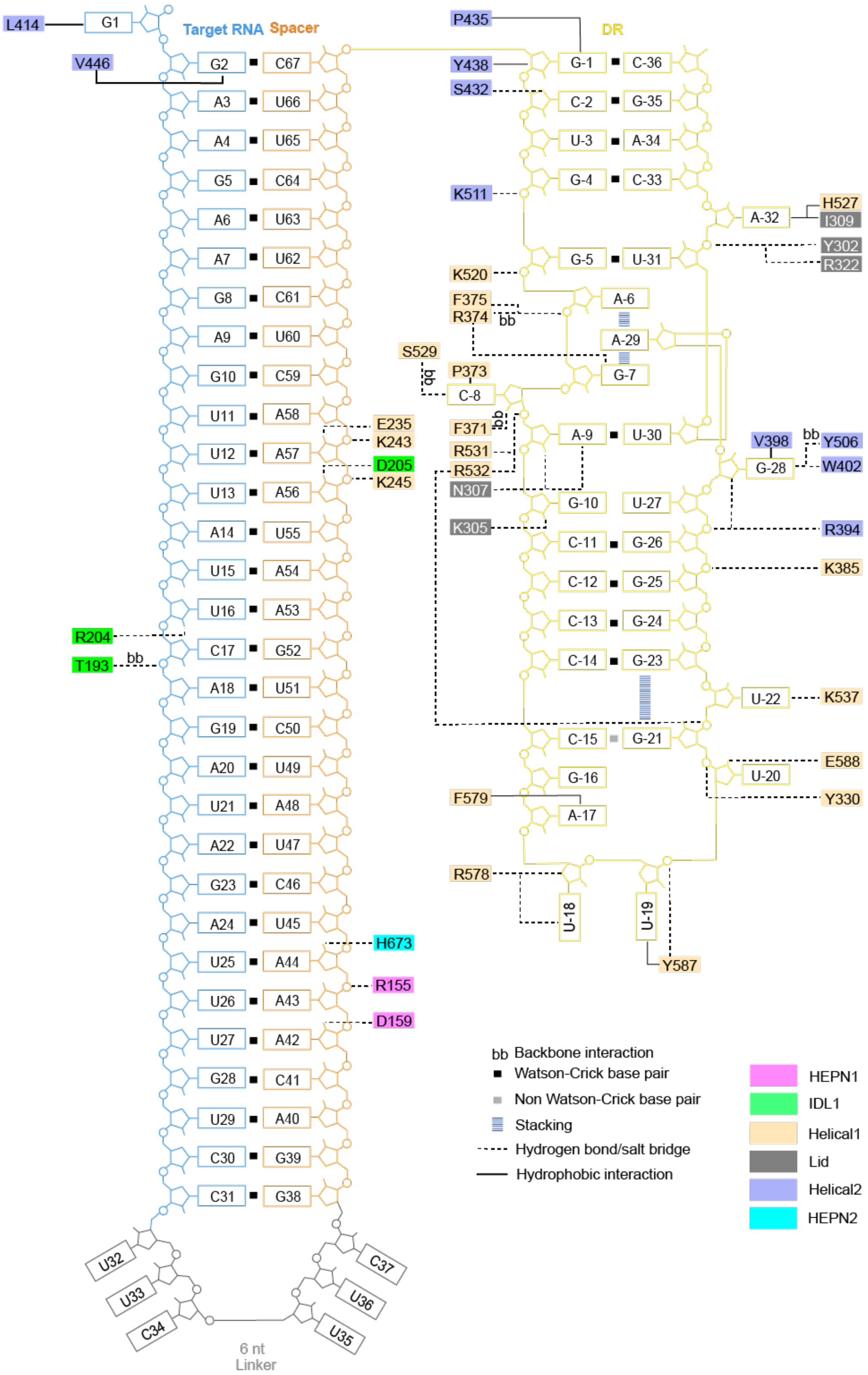


**Supplementary Fig. 5 Schematics of protein-RNA interactions in Cas13bt3$^{Act}$.** The 6 nt linker between spacer and target is disorder and colored grey. bb, backbone interaction.

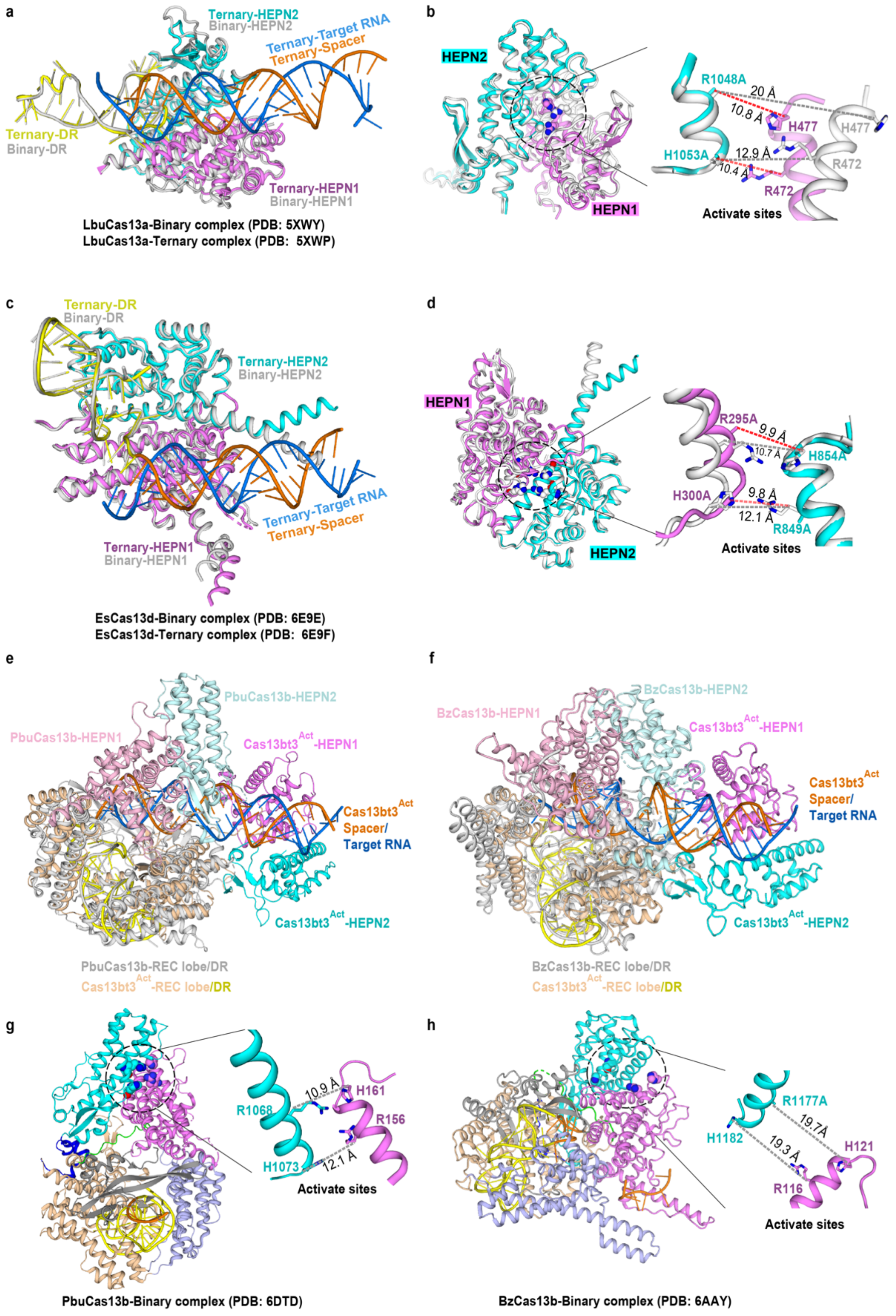


**Supplementary Fig. 6 Structural comparisons of LbuCas13a, EsCas13d, PbuCas13b and BzCas13b.**

**a** Structural alignment of binary and ternary complexes of LbuCas13a. **b** Zoom-in views of the HEPN domains in binary and ternary complexes of LbuCas13a. **c** Structural alignment of binary

and ternary complexes of EsCas13d. **d** Zoom-in views of the HEPN domains in binary and ternary complexes of EsCas13d. (**a, c**) The DR RNA was aligned and only NUC lobe was shown for clarity. **e-f** Structural alignment of PbuCas13b (**e**) or BzCas13b (**f**) with Cas13bt3$^{Act}$, the REC lobes were aligned in both (**e**) and (**f**). **g-h** The binary structure of PbuCas13a (**g**) and BzCas13b (**h**), The active sites' distance between Calpha-atoms of the in both structures are indicated.

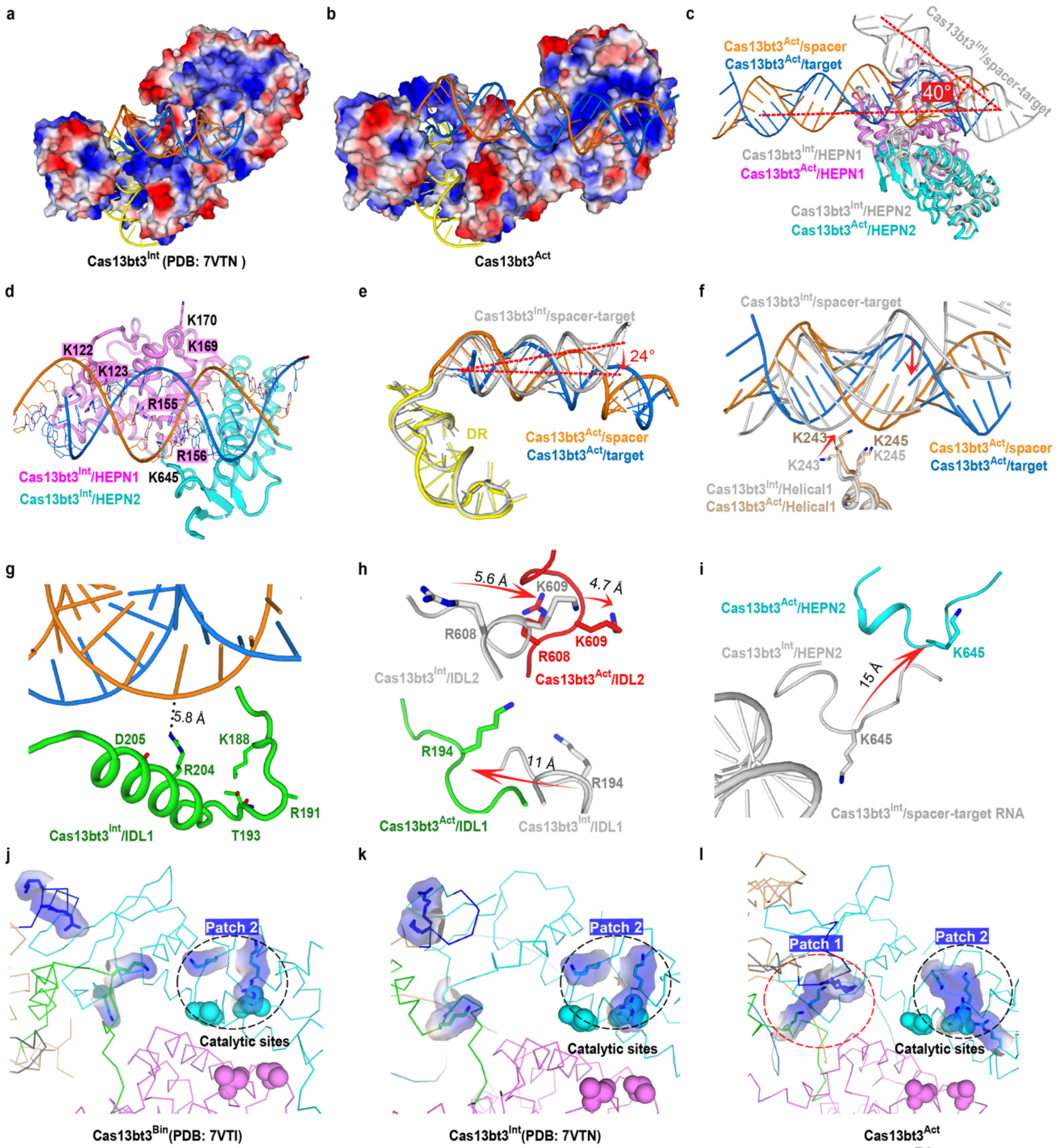


**Supplementary Fig. 7 The RNA binding interface comparisons of Cas13bt3$^{Bin}$, Cas13bt3$^{Int}$ and Cas13bt3$^{Act}$.**

**a-b** The electrostatic potential surface of Cas13bt3$^{Int}$ and Cas13bt3$^{Act}$ **c** Structural alignment of HEPN domains and spacer/target RNAs in both Cas13bt3$^{Int}$ (grey) and Cas13bt3$^{Act}$ (colored). The dsRNA tilted 40 degrees from Cas13bt3$^{Int}$ to Cas13bt3$^{Act}$ when the HEPN domains are aligned. **d** The possible RNA interacting residues in Cas13bt3$^{Int}$. **e** Structural alignment of spacer/target duplex RNA in Cas13bt3$^{Int}$ and Cas13bt3$^{Act}$ with the DR aligned. **f** The structural comparison of Helical 1 loop in Cas13bt3$^{Int}$ and Cas13bt3$^{Act}$, the conformational change of K243 and the spacer/target duplex are indicated with red arrow. **g** Zoom-in view of IDL1 and the spacer/target duplex in Cas13bt3$^{Int}$, the distance between R204 and dsRNA is indicated. **h-i** The structural comparison of R608, K609 on IDL2, R194 on IDL1, and K645 on HEPN2 in Cas13bt3$^{Int}$ and Cas13bt3$^{Act}$, the movement of each residue is indicated with red arrow. **j-l** The structure difference of Patch 1 composed residues in Cas13bt3$^{Bin}$, Cas13bt3$^{Int,}$ and Cas13bt3$^{Act}$,

Patch 1 only formed upon the long target RNA binding in the active state. Color codes are defined as in Fig. 1c and 1d, respectively.

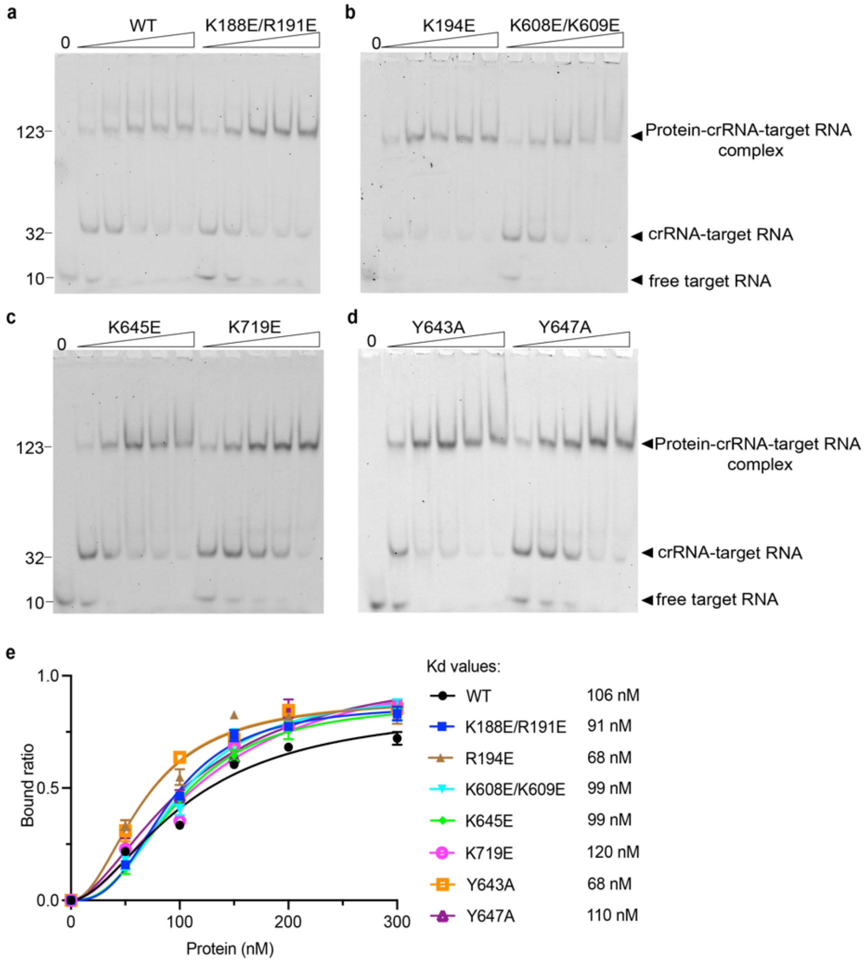


**Supplementary Fig. 8 Quantification of target RNA binding affinity with WT and mutated Cas13bt3.**

**a-d** Representative EMSA gel images of WT and mutant Cas13bt3 complex with crRNA binding to 10 nM of 5'-FAM-T1. The protein-crRNA complex at 1:1 molar ratio was serially diluted to 300, 200, 150, 100, and 50 nM. **e** Calculation of binding affinity between protein-crRNA and target RNA. Bound and unbound fractions were quantified by densitometry and fitted. (**a-d**) RNA or protein-RNA complex sizes (in kDa) are indicated. At least two times each experiment was repeated independently with similar results. (**e**) Data are present as means ± SDs (n=2). Source data are provided as Source Data File.

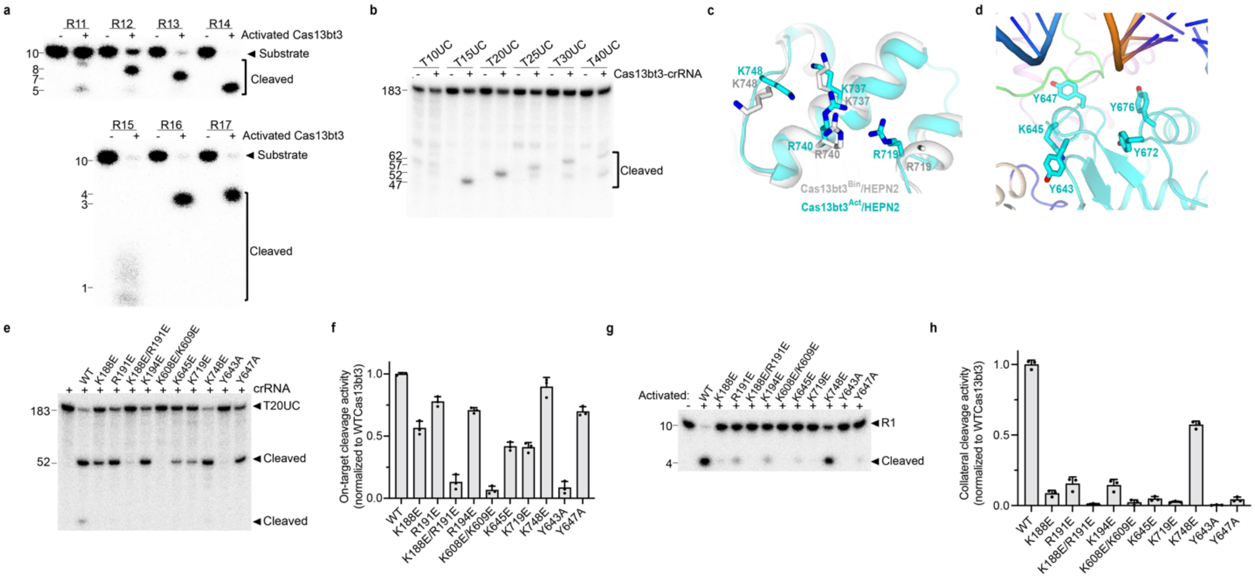

**Supplementary Fig. 9 Substrate length preference of Cas13bt3 and engineering of Cas13bt3 for high-fidelity cleavage.**

**a** Representative gels of the collateral cleavage activities of Cas13bt3 with UC site at different positions on ssRNA substrates. **b** Representative gels of the on-target RNA cleavage activities of Cas13bt3 with UC site at the different positions on target RNA. **c** Structural alignment of positively charged residues on Pacth2 in Cas13bt3$^{Bin}$ and Cas13bt3$^{Act}$. **d** The relative positions of Y643, K645, Y647, and Y672/Y676A in Cas13bt3$^{Act}$. Color codes are defined as in Fig. 1c and 1d. **e** Representative gels of on-target cleavage activities of WT and mutated Cas13bt3. **f** Normalized on-target cleavage activity as shown in (**e**). **g** Representative gels of collateral cleavage activities of WT and mutated Cas13bt3. **h** Normalized collateral cleavage activity as shown in (**g**). The target RNA (T20UC) was used as the substrate for on-target cleavage assay and the reporter RNA (R1) was used as the substrate for collateral cleavage assay. (**a**, **b**, **e**, **g**) RNA sizes (in nt) are indicated. At least three times each experiment was repeated independently with similar results. (**f**, **h**) Data are present as means ± SDs (n=3). Source data are provided as Source Data File.

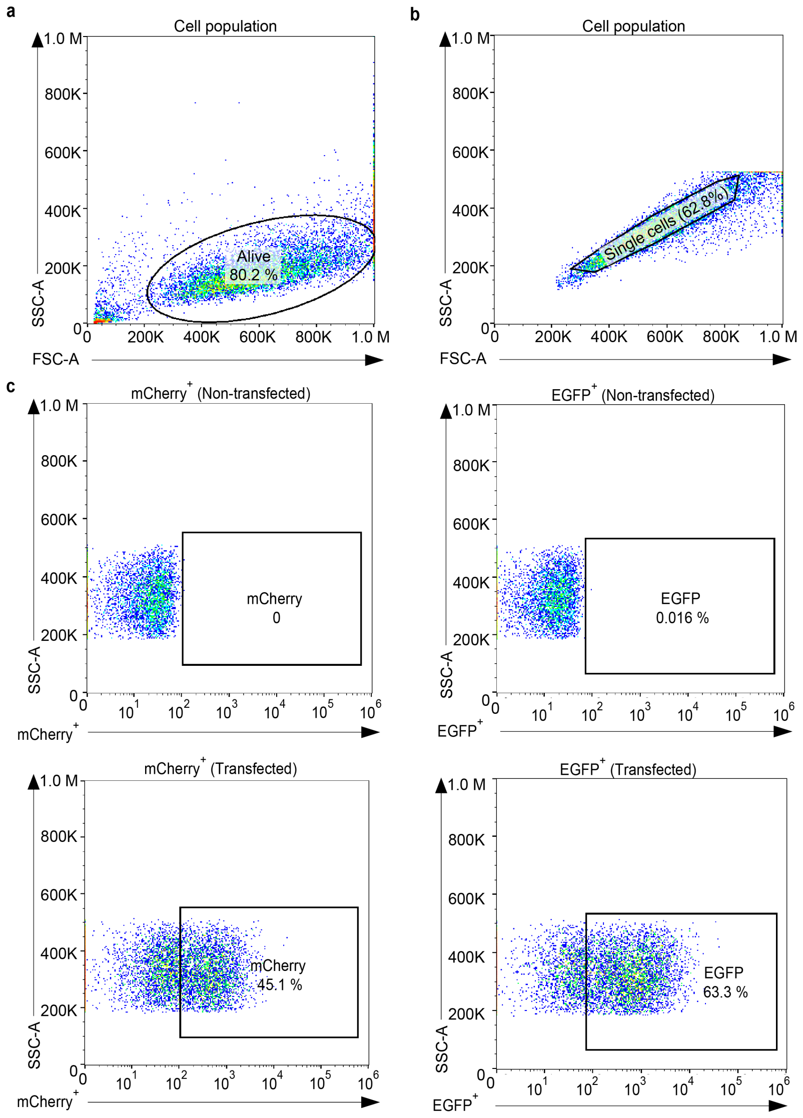


**Supplementary Fig. 10 Gating strategy for flow cytometry experiments.**
**a-b** An example well is gated by collecting the live cell population (**a**) and then stratifying the resulting population by FSC-A and FSC-H to only keep single cells (**b**). **c** The proportion of mCherry+ or EGFP+ is captured for a non-transfected well, and a transfected well. Negative cells are those captured outside of their respective boxes in the non-transfected condition, while positive is within the bounds.

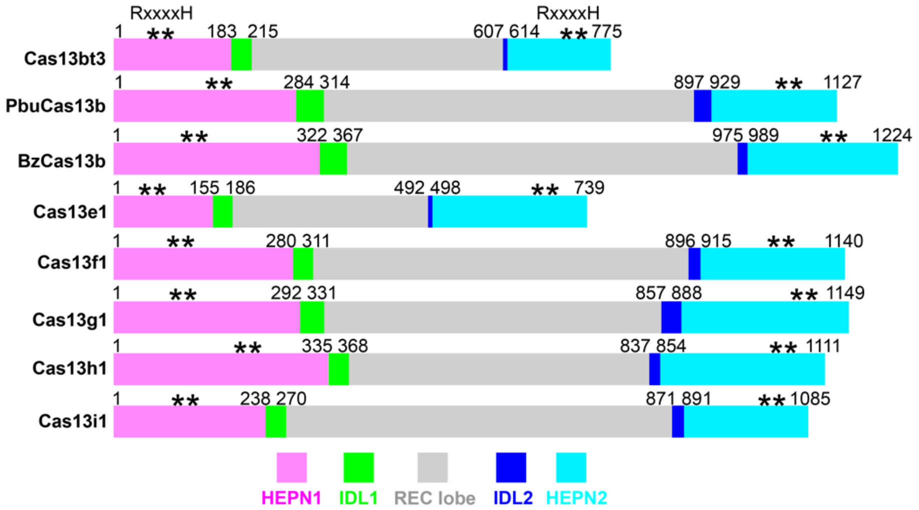


**Supplementary Fig. 11 Domain organization of newly identified Cas13 effectors.** The domains assignments of Cas13e1-Cas13i1 are based on sequence alignment with Cas13bt3 or PbuCas13b and Alpha-fold predicted structures. RxxxxH: the activate sites motif of HEPN domains.

**Supplementary Table 1**

**Cryo-EM data collection, model refinement, and validation for Cas13bt3$^{Act}$**

| | |
|---|---|
| **Data collection and processing** | |
| EMDB ID | EMD-29433 |
| PDB ID | 8FTI |
| Microscope | Titan Krios G3 |
| Detector | Gatan K2 camera |
| Magnification | 165000 |
| Voltage (kV) | 300 |
| Electron exposure (e-/ Å$^2$) | 49 |
| Defocus range (μM) | -0.6 to -2.2 μM |
| Pixel size (Å) | 0.435 |
| Number of movies | 5594 |
| Initial particles (no.) | 12310532 |
| Final particles (no.) | 37228 |
| Map resolution (Å) | 3.5 |
| FSC threshold | 0.143 |
| Map sharpening B factor (Å2) | -101.4 |
| **Refinement** | |
| No. of atoms | 8126 |
| No. of residues | |
| Protein | 737 |
| Nucleic acid | 97 |
| R.m.s. deviations | |
| Bond lengths (Å) | 0.002 |
| Bond angles (°) | 0.558 |
| **Validation** | |
| Clashscore | 9.2 |
| Rotamer outlier (%) | 4.55 |
| Ramachandran plot (%) | |
| Favored | 95.47 |
| Allowed | 4.53 |
| Outliers | 0 |

## Supplementary Table 2

### Oligonucleotides for Cas13bt3 cloning

| Mutation (for in vitro studies) | Forward primer | Reverse primer |
|---|---|---|
| A84RA89H | CGCAACTACTTTTCCCACTACAGGCACAGCCCTGGCTGC | CAGGGCCTCGGCTTTGGCATTG |
| A739RA744H | CGCAGAGCCTTTTTCCACCACCACCTGAAATTTGTGATT | CACCTTATTCACGGCGGTCTTC |
| K54A | ACACGAGGCATATTCCAAGCACGACTGGTAC | GGAATATGCCTCGTGTCTCAGCAGG GACTCC |
| K54E | CACGAGGAATATTCCAAGCACGACTGGTACGAT | GGAATATTCCTCGTGTCTCAGCAGGG ACT |
| K57A | ATATTCCGCGCACGACTGGTACGATGAGGAT | GTCGTGCGCGGAATATTTCTCGTGTC TCAGC |
| R155A/R156A | TGGAGGCGGCGGTGCTGGACAGGCTGTATGG | AGCACCGCCGCCTCCACGAAGAAGG ACACGA |
| R179A | ACTGACCGCAAAGGCCCTGTCCATGTACTGT | GGCCTTTGCGGTCAGTTTGTACTGG CCCTCG |
| R179E/K180E | ACCGAAGAAGCCCTGTCCATGTACTGTCTGA | CAGGGCTTCTTCGGTCAGTTTGTACT GGCCCTC |
| K188E | CTGTCTGGAAGACAGCAGGTTTACCAAGGCC | TGCTGTCTTCCAGACAGTACATGGAC AGGGCC |
| R191E | GGACAGCGAGTTTACCAAGGCCTGGGATAAGAGG | TGGTAAACTCGCTGTCCTTCAGACAG TACATGGA |
| K188E/R191E | CTGGAAGACAGCGAATTTACCAAGGCCTGGGATAAGAGG | AAATTCGCTGTCTTCCAGACAGTACA TGGACAGGGCC |
| K194E | GTTTACCGAAGCCTGGGATAAGAGGGTGCTG | CCCAGGCTTCGGTAAACCTGCTGTC CTTCAGACA |
| K243E/K245E | ACGAGGATGAATTTATCGAGTTTGCCCTGCACTAC | TAAATTCATCCTCGTGTCTCTTAGGAT TGGTGCCCT |
| R204E | CTGTTTGAAGACATCCTGGCCCAGCTG | GATGTCTTCAAACAGCAGCACCCTCT TATCC |
| R608E/K609E | GCGTGGAAGAGGAGCTGGGCAATAAGATTGTGTGG | GCTCCTCTTCCACGCTGCCCAGAAA ATACTGG |
| Y643A | GTCCGACGCCGGCAAACTGTACGTGCTGGAC | GTTTGCCGGCGTCGGACACGCTGAA CACAAT |
| K645E | CTATGGCGAGCTGTACGTGCTGGACGACG | CGTACAGCTCGCCATAGTCGGACAC GCTG |
| Y647A | CAAACTGGCCGTGCTGGACGACGCCG | CCAGCACGGCCAGTTTGCCATAGTC GGACACG |
| R681A | GGGCTTTGCAGCCTACAACGACCTGCAGAAG | GTAGGCTGCAAAGCCCTTCTCGTACA CGGTG |
| R719A | GACTTCGCAGAGATCCTGGCCCAAACCATGTG | GATCTCTGCGAAGTCGATGTAGTGG GCGCC |
| R719E | CGACTTCGAGGAGATCCTGGCCCAAACCATGT | GGATCTCCTCGAAGTCGATGTAGTGG GCGC |
| K748E | CCACCTGGAGTTTGTGATTGACGAGTTTGGCCTG | TCACAAACTCCAGGTGGTGGTGGAA AAAGGC |
| **Mutation (for in vivo studies)** | **Forward primer** | **Reverse primer** |
| R84A/H89A | GCCAACTACTTCAGTGCCTACCGGCATAGCCCTGGCTG | GGCACTGAAGTAGTTGGCCAGGGCT TCAGCCTTGGCG |
| R739A/H744A | GCTCGCGCCTTCTTCGCTCACCACCTGAAGTTCGTGATT GACG | GAAGAAGGCGCGAGCCACCTTGTTC ACTGCGGTCTTTTCG |
| Y643A | TCTGACGCTGGCAAGCTGTACGTGCTGGAC | CTTGCCAGCGTCAGACACGGAGAAC ACGATGC |
| Y647A | CAAGCTGGCCGTGCTGGACGATGCCGAATTCC | AGCACGGCCAGCTTGCCGTAGTCAG ACACGG |
| Cas13bt3_ AgeI_F | TTCAGGTTGGACCGGTGCCACCATGCCCAAGAAGAAGC GGAAGGTGGCCCAGGTGAGCAAGCAGA | |
| Cas13bt3_ HindIII_R | TAAACAAGTTAAGCTTCTACACCTTCCGCTTCTTCTTGGG CTTGACAGGGAACTTCC | |

**Supplementary Table 3**

**The in-vitro RNA transcription template used in this study**

| Transcription template | Forward primer | Reverse primer |
|---|---|---|
| hpIVT-S1 | AGCTTAATACGACTCACTATAGGAAGAAGAG | TAAACTCTTCTTCCTATAGTGAGTCGTATTA |
| hpIVT-S2 | TTTATTCAGATAGATTTGTCCTTCTTCGGA | TTTGTCCGAAGAAGGACAAATCTATCTGAA |
| hpIVT-S3 | CAAATCTATCTGAATAAACTCTTCTTCGCT | CTCCAGCGAAGAAGAGTTTATTCAGATAGA |
| hpIVT-S4 | GGAGCAGCCCCCGATTTGTGGGGTGATTACAGC | CTAGGCTGTAATCACCCCACAAATCGGGGGCTG |
| M13F | GAGCGGATAACAATTTCACACAGGAAAC | |
| hpIVT-R | | GCTGTAATCACCCCACAAAT |
| T0-S1 | AGCTTAATACGACTCACTATAGGAAGAAGAGTTTAT | CTGAATAAACTCTTCTTCCTATAGTGAGTCGTATTA |
| T0-S2 | TCAGATAGATTTGTCCGCTCGACCTTTTTAGGTCGA | CGACTCGACCTAAAAAGGTCGAGCGGACAAATCTAT |
| T0-S3 | GTCGACGGAGTCTAGACTCCGTCCTGATGAGTCCGT | CCTCACGGACTCATCAGGACGGAGTCTAGACTCCGT |
| T0-S4 | GAGGACGAAAGACTCGACCTGATGTATAGAGTAGA | CTAGTCTACTCTATACATCAGGTCGAGTCTTTCGT |
| T0-R | | TCTACTCTATACATCAGGTCGAG |
| T10UC/T15UC/T20UC/T25UC/T30UC/T40UC-S1 | AGCTTAATACGACTCACTATAGGAAGAAGAGTTTATTCAGATAGATTTGTCC | GTGCGGACAAATCTATCTGAATAAACTCTTCTTCCTATAGTGAGTCGTATTA |
| T10UC-S2 | GCACGACCAATCTAAAACAAGTACACACAAGAGACTAGACACACACACACTG | TCATCAGTGTGTGTGTGTCTAGTCTCTTGTGTGTACTTGTTTTAGATTGGTC |
| T10UC/T15UC/T20/T25UC/T30UC/T40UC/-S3 | ATGAGACCACCAGAGACAGAAAGACTACAGACAATAGATAGATATAGACAGA | ATTTTCTGTCTATATCTATCTATTGTCTGTAGTCTTTCTGTCTCTGGTGGTC |
| T10UC/T15UC/T20UC/T25UC/T30UC/T40UC-S4 | AAATGAAGCACATGTAAATAAGATGCAACAACAGTAAGCATATAAATG | CTAGCATTTATATGCTTACTGTTGTTGCATCTTATTTACATGTGCTTC |
| T15UC-S2 | GCACGACCAATAAAATCCAAGTACACACAAGAGACTAGACACACACACACTG | TCATCAGTGTGTGTGTGTCTAGTCTCTTGTGTGTACTTGGATTTTATTGGTC |
| T20UC-S2 | GCACGACCAATAAAACAAGTTCACACACAAGAGACTAGACACACACACACTG | TCATCAGTGTGTGTGTGTCTAGTCTCTTGTGTGTGAACTTGTTTTATTGGTC |
| T25UC-S2 | GCACGACCAATAAAACAAGTACACATCCAAGAGACTAGACACACACACACTG | TCATCAGTGTGTGTGTGTCTAGTCTCTTGGATGTGTACTTGTTTTATTGGTC |
| T30UC-S2 | GCACGACCAATAAAACAAGTACACACAAGATCGACTAGACACACACACACTG | TCATCAGTGTGTGTGTGTCTAGTCGATCTTGTGTGTACTTGTTTTATTGGTC |
| T40UC-S2 | GCACGACCAATAAAACAAGTACACACAAGAGACTAGACACTCACACACACTG | TCATCAGTGTGTGTGTGTCTAGTCGATCTTGTGTGTACTTGTTTTATTGGTC |
| TUC-R | | CATTTATATGCTTACTGTTGTTGCATCTTATTTACATGT |
| crIVT | TAATACGACTCACTATAGGACAAATCTATCTGAATAAACTCTTCTTCGCTGGAGCAGCCCCCGATTTGTGGGGTGATTACAGC | GCTGTAATCACCCCACAAATCGGGGGCTGCTCCAGCGAAGAAGAGTTTATTCAGATAGATTTGTCCTATAGTGAGTCGTATTA |
| T1IVT | TAATACGACTCACTATAGGAAGAAGAGTTTATTCAGATAGATTTGTCCG | CGGACAAATCTATCTGAATAAACTCTTCTTCCTATAGTGAGTCGTATTA |
| T2IVT | TAATACGACTCACTATAGGAAGAAGAGTTTATTCAGATAGATTTGG | CCAAATCTATCTGAATAAACTCTTCTTCCTATAGTGAGTCGTATTA |
| T3IVT | TAATACGACTCACTATAGGAAGAAGAGTTTATTCAGATAGATG | CATCTATCTGAATAAACTCTTCTTCCTATAGTGAGTCGTATTA |
| T4IVT | TAATACGACTCACTATAGGAAGAAGAGTTTATTCAGATAC | GTATCTGAATAAACTCTTCTTCCTATAGTGAGTCGTATTA |

| | | |
|---|---|---|
| T5IVT | TAATACGACTCACTATAGGAAGAAGAGTTTATTCAGATAGATTTGTCAA | TTGACAAATCTATCTGAATAAACTCTTCTTCCTATAGTGAGTCGTATTA |
| T6IVT | TAATACGACTCACTATAGGAAGAAGAGTTTATTCAGATAGATTTGTAAA | TTTACAAATCTATCTGAATAAACTCTTCTTCCTATAGTGAGTCGTATTA |
| T7IVT | TAATACGACTCACTATAGGAAGAAGAGTTTATTCAGATAGATTTGAAAA | TTTTCAAATCTATCTGAATAAACTCTTCTTCCTATAGTGAGTCGTATTA |
| T8IVT | TAATACGACTCACTATAGGAAGAAGAGTTTATTCAGATAGATTTAAAAA | TTTTTAAATCTATCTGAATAAACTCTTCTTCCTATAGTGAGTCGTATTA |
| T9IVT | TAATACGACTCACTATAGGAAGAAGAGTTTATTCAGATAGATTAAAAAA | TTTTTTAATCTATCTGAATAAACTCTTCTTCCTATAGTGAGTCGTATTA |
| T10IVT | TAATACGACTCACTATAGGAAGAAGAGTTTATTCAGATAGATAAAAAAA | TTTTTTTATCTATCTGAATAAACTCTTCTTCCTATAGTGAGTCGTATTA |
| T11IVT | TAATACGACTCACTATAGGAAGAAGAGTTTATTCAGATAGAAAAAAAAA | TTTTTTTTTCTATCTGAATAAACTCTTCTTCCTATAGTGAGTCGTATTA |
| T12IVT | TAATACGACTCACTATAGGTAAGAAGAGTTTATTCAGATAGATTTGTCCG | CGGACAAATCTATCTGAATAAACTCTTCTTACCTATAGTGAGTCGTATTA |
| T13IVT | TAATACGACTCACTATAGGTTAGAAGAGTTTATTCAGATAGATTTGTCCG | CGGACAAATCTATCTGAATAAACTCTTCTAACCTATAGTGAGTCGTATTA |
| T14IVT | TAATACGACTCACTATAGGTTTGAAGAGTTTATTCAGATAGATTTGTCCG | CGGACAAATCTATCTGAATAAACTCTTCAAACCTATAGTGAGTCGTATTA |
| T15IVT | TAATACGACTCACTATAGGTTTTAAGAGTTTATTCAGATAGATTTGTCCG | CGGACAAATCTATCTGAATAAACTCTTAAAACCTATAGTGAGTCGTATTA |
| T16IVT | TAATACGACTCACTATAGGTTTTTAGAGTTTATTCAGATAGATTTGTCCG | CGGACAAATCTATCTGAATAAACTCTAAAAACCTATAGTGAGTCGTATTA |
| T17IVT | TAATACGACTCACTATAGGTTTTTTGAGTTTATTCAGATAGATTTGTCCG | CGGACAAATCTATCTGAATAAACTCAAAAAACCTATAGTGAGTCGTATTA |
| T18IVT | TAATACGACTCACTATAGGAAGAAGAGTTTATTCAGATAGATTTCACCG | CGGTGAAATCTATCTGAATAAACTCTTCTTCCATAGTGAGTCGTATTA |
| T19IVT | TAATACGACTCACTATAGGAAGAAGAGTTTATTCAGATAGTATTGTCCG | CGGACAATACTATCTGAATAAACTCTTCTTCCATAGTGAGTCGTATTA |
| T20IVT | TAATACGACTCACTATAGGAAGAAGAGTTTATTCAGTAAGATTTGTCCG | CGGACAAATCTTACTGAATAAACTCTTCTTCCATAGTGAGTCGTATTA |
| T21IVT | TAATACGACTCACTATAGGAAGAAGAGTTTATAGAGATAGATTTGTCCG | CGGACAAATCTATCTCTATAAACTCTTCTTCCATAGTGAGTCGTATTA |
| T22IVT | TAATACGACTCACTATAGGAAGAAGAGTAAATTCAGATAGATTTGTCCG | CGGACAAATCTATCTGAATTTACTCTTCTTCCTATAGTGAGTCGTATTA |
| T23IVT | TAATACGACTCACTATAGGAAGAATTGTTTATTCAGATAGATTTGTCCG | CGGACAAATCTATCTGAATAAACAATTCTTCCTATAGTGAGTCGTATTA |

## Supplementary Table 4

### The RNAs used in this study

| RNA | Sequence (the guide/target sequences are underlined, the UC cleavage site are colored red, the mismatch are indicated with lower case letters ) |
|---|---|
| crRNA | GGACAAAUCUAUCUGAAUAAACUCUUCUUCGCUGGAGCAGCCCCCGAUUUGUGGGGUGAUUACAGC |
| hpRNA | GGAAGAAGAGUUUAUUCAGAUAGAUUUGUCCUUCUUCGGACAAAUCUAUCUGAAUAAACUCUUCUUCGCUGGAGCAGCCCCCGAUUUGUGGGGUGAUUACAGC |
| T0 | GGAAGAAGAGUUUAUUCAGAUAGAUUUGUCCGCUCGACCUUUUUAGGUCGAGUCGACGGAGUCUAGACUCCGUCCUGAUGAGUCCGUGAGGACGAAAGACUCGACCUGAUGUAUAGAGUAGA |
| T10UC | GGAAGAAGAGUUUAUUCAGAUAGAUUUGUCCGCACGACCAAUCUAAAACAAGUACACACAAGAGACUAGACACACACACACUGAUGAGACCACCAGAGACAGAAAGACUACAGACAAUAGAUAGAUAUAGACAGAAAAUGAAGCACAUGUAAAUAAGAUGCAACAACAGUAAGCAUAUAAAUG |
| T15UC | GGAAGAAGAGUUUAUUCAGAUAGAUUUGUCCGCACGACCAAUAAAAUCCAAGUACACACAAGAGACUAGACACACACACACUGAUGAGACCACCAGAGACAGAAAGACUACAGACAAUAGAUAGAUAUAGACAGAAAAUGAAGCACAUGUAAAUAAGAUGCAACAACAGUAAGCAUAUAAAUG |
| T20UC | GGAAGAAGAGUUUAUUCAGAUAGAUUUGUCCGCACGACCAAUAAAACAAGUUCACACACAAGAGACUAGACACACACACACUGAUGAGACCACCAGAGACAGAAAGACUACAGACAAUAGAUAGAUAUAGACAGAAAAUGAAGCACAUGUAAAUAAGAUGCAACAACAGUAAGCAUAUAAAUG |
| T25UC | GGAAGAAGAGUUUAUUCAGAUAGAUUUGUCCGCACGACCAAUAAAACAAGUACACAUCCAAGAGACUAGACACACACACACUGAUGAGACCACCAGAGACAGAAAGACUACAGACAAUAGAUAGAUAUAGACAGAAAAUGAAGCACAUGUAAAUAAGAUGCAACAACAGUAAGCAUAUAAAUG |
| T30UC | GGAAGAAGAGUUUAUUCAGAUAGAUUUGUCCGCACGACCAAUAAAACAAGUACACACAAGAUCGACUAGACACACACACACUGAUGAGACCACCAGAGACAGAAAGACUACAGACAAUAGAUAGAUAUAGACAGAAAAUGAAGCACAUGUAAAUAAGAUGCAACAACAGUAAGCAUAUAAAUG |
| T40UC | GGAAGAAGAGUUUAUUCAGAUAGAUUUGUCCGCACGACCAAUAAAACAAGUACACACAAGAGACUAGACACUCACACACACUGAUGAGACCACCAGAGACAGAAAGACUACAGACAAUAGAUAGAUAUAGACAGAAAAUGAAGCACAUGUAAAUAAGAUGCAACAACAGUAAGCAUAUAAAUG |
| T1 | GGAAGAAGAGUUUAUUCAGAUAGAUUUGUCCG |
| T2 | GGAAGAAGAGUUUAUUCAGAUAGAUUUGG |
| T3 | GGAAGAAGAGUUUAUUCAGAUAGAUG |
| T4 | GGAAGAAGAGUUUAUUCAGAUAC |
| T5 | GGAAGAAGAGUUUAUUCAGAUAGAUUUGUCAA |
| T6 | GGAAGAAGAGUUUAUUCAGAUAGAUUUGUAAA |
| T7 | GGAAGAAGAGUUUAUUCAGAUAGAUUUGAAAA |
| T8 | GGAAGAAGAGUUUAUUCAGAUAGAUUUAAAAA |
| T9 | GGAAGAAGAGUUUAUUCAGAUAGAUUAAAAAA |
| T10 | GGAAGAAGAGUUUAUUCAGAUAGAUAAAAAAA |
| T11 | GGAAGAAGAGUUUAUUCAGAUAGAAAAAAAAA |
| T12 | GGUAAGAAGAGUUUAUUCAGAUAGAUUUGUCCG |
| T13 | GGUUAGAAGAGUUUAUUCAGAUAGAUUUGUCCG |
| T14 | GGUUUGAAGAGUUUAUUCAGAUAGAUUUGUCCG |
| T15 | GGUUUUAAGAGUUUAUUCAGAUAGAUUUGUCCG |
| T16 | GGUUUUUAGAGUUUAUUCAGAUAGAUUUGUCCG |
| T17 | GGUUUUUUGAGUUUAUUCAGAUAGAUUUGUCCG |
| T18 | GGAAGAAGAGUUUAUUCAGAUAGAUUUcaCCG |
| T19 | GGAAGAAGAGUUUAUUCAGAUAGuaUUGUCCG |
| T20 | GGAAGAAGAGUUUAUUCAGuaAGAUUUGUCCG |
| T21 | GGAAGAAGAGUUUAUagAGAUAGAUUUGUCCG |
| T22 | GGAAGAAGAGUaaAUUCAGAUAGAUUUGUCCG |
| T23 | GGAAGAAuuGUUUAUUCAGAUAGAUUUGUCCG |
| 5'-FAM-ssRNA36 | 5'-6-FAM-ACCCCGCAUUACGUUUGGUGGACCCUCAGAUUCAAC |
| 5'-FAM-U20 | 5'-6-FAM-UUUUUUUUUUUUUUUUUUUU |
| R1 | UGUUCGACGA |
| 5'-FAM-R1 | 5'-6-FAM-UGUUCGACGA |

| | |
|---|---|
| R1-FQ | 5'-6-FAM-UGUUCGACGA-IABkFQ-3' |
| R2 | UGAUCGACGA |
| R3 | UGUACGACGA |
| R4 | UGUUAGACGA |
| R5 | UGACUGACGA |
| R6 | UGAUCUACGA |
| R7 | UGAUCAACGA |
| R8 | UGAUCCACGA |
| R9 | UGCUCGACGA |
| R10 | UGGUCGACGA |
| R11 | UUUUUUGAUC |
| R12 | UUUUUGAUCG |
| R13 | UUUUGAUCGA |
| R14 | UUGAUCGACG |
| R15 | UCAGUUUUUU |
| R16 | GAUCAGUUUU |
| R17 | GCGAUCAGUU |
| A10/AA10 | AAAAAAAAAA |
| C10 | CCCCCCCCCC |
| G10 | GGGGGGGGGG |
| U10 | UUUUUUUUUU |
| AC10 | AAAACAAAAA |
| AG10 | AAAAGAAAAA |
| AU10 | AAAAUAAAAA |
| CA10 | AAACAAAAAA |
| CC10 | AAACCAAAAA |
| CG10 | AAACGAAAAA |
| CU10 | AAACUAAAAA |
| GA10 | AAAGAAAAAA |
| GC10 | AAAGCAAAAA |
| GG10 | AAAGGAAAAA |
| GU10 | AAAGUAAAAA |
| UA10 | AAAUAAAAAA |
| UC10 | AAAUCAAAAA |
| UG10 | AAAUGAAAAA |
| UU10 | AAAUUAAAAA |
