# Supplementary material for: Structural basis for the activation of a compact CRISPR-Cas13 nuclease
Source: Nat Commun. 2023 Sep 20;14:5845. doi: 10.1038/s41467-023-41501-5 (PMC10511502; doi:10.1038/s41467-023-41501-5)
Supplement: Supplementary file 3 — Reporting Summary [file 41467_2023_41501_MOESM3_ESM.pdf]

nature portfolio

Corresponding author(s): Yang Gao
Last updated by author(s): Aug 10, 2023

Reporting Summary

Nature Portfolio wishes to improve the reproducibility of the work that we publish. This form provides structure for consistency and transparency in reporting. For further information on Nature Portfolio policies, see our Editorial Policies and the Editorial Policy Checklist.

Statistics

For all statistical analyses, confirm that the following items are present in the figure legend, table legend, main text, or Methods section.

n/a	Confirmed	
☐	☒	The exact sample size (*n*) for each experimental group/condition, given as a discrete number and unit of measurement
☐	☒	A statement on whether measurements were taken from distinct samples or whether the same sample was measured repeatedly
☒	☐	The statistical test(s) used AND whether they are one- or two-sided<br>*Only common tests should be described solely by name; describe more complex techniques in the Methods section.*
☒	☐	A description of all covariates tested
☒	☐	A description of any assumptions or corrections, such as tests of normality and adjustment for multiple comparisons
☐	☒	A full description of the statistical parameters including central tendency (e.g. means) or other basic estimates (e.g. regression coefficient) AND variation (e.g. standard deviation) or associated estimates of uncertainty (e.g. confidence intervals)
☒	☐	For null hypothesis testing, the test statistic (e.g. *F*, *t*, *r*) with confidence intervals, effect sizes, degrees of freedom and *P* value noted<br>*Give P values as exact values whenever suitable.*
☒	☐	For Bayesian analysis, information on the choice of priors and Markov chain Monte Carlo settings
☒	☐	For hierarchical and complex designs, identification of the appropriate level for tests and full reporting of outcomes
☒	☐	Estimates of effect sizes (e.g. Cohen's *d*, Pearson's *r*), indicating how they were calculated

Our web collection on statistics for biologists contains articles on many of the points above.

Software and code

Policy information about availability of computer code

Data collection	The cryo-EM data is collected with a K2 Summit camera through SeriaEl M software (3.8). The cryo-EM data was processed through RELION (4.0.beat) and cryoSPARC(3. 3.2). The initial structure prediction was performed with Alpha Fold Colab sever. The flow cytometry data were collected by using a SONY SA3800.
Data analysis	Software COOT (0.9.6), PHENIX (1.20.1), pymol (2.2.0), and Chimera (1.14) were used for data analysis, structural refinement, and structural illustration. The flow cytometry data were analyzed using FlowJo (10.4.0). Statistical tests were performed using Prism (9 .4. 1) .

For manuscripts utilizing custom algorithms or software that are central to the research but not yet described in published literature, software must be made available to editors and reviewers. We strongly encourage code deposition in a community repository (e.g. GitHub). See the Nature Portfolio guidelines for submitting code & software for further information.

Data

Policy information about availability of data

All manuscripts must include a data availability statement. This statement should provide the following information, where applicable:

- Accession codes, unique identifiers, or web links for publicly available datasets
- A description of any restrictions on data availability
- For clinical datasets or third party data, please ensure that the statement adheres to our policy

All original data and materials will be available upon request. The three-dimensional cryo-EM density maps for Cas13bt3Act complexes have been deposited in the EM Database under the accession code EMDB: EMD-29433 [https://www.ebi.ac.uk/pdbe/entry/emdb/EMD-29433], and the coordinates for the structure have

been deposited in Protein Data Bank under accession code PDB 8FTI [https://doi.org/10.2210/pdb8FTI/pdb]. Source data are provided with this paper.

Research involving human participants, their data, or biological material

Policy information about studies with human participants or human data. See also policy information about sex, gender (identity/presentation), and sexual orientation and race, ethnicity and racism.

Reporting on sex and gender	Not applicable.
Reporting on race, ethnicity, or other socially relevant groupings	Not applicable.
Population characteristics	Not applicable.
Recruitment	Not applicable.
Ethics oversight	Not applicable.

Note that full information on the approval of the study protocol must also be provided in the manuscript.

Field-specific reporting

Please select the one below that is the best fit for your research. If you are not sure, read the appropriate sections before making your selection.

☒ Life sciences ☐ Behavioural & social sciences ☐ Ecological, evolutionary & environmental sciences

For a reference copy of the document with all sections, see nature.com/documents/nr-reporting-summary-flat.pdf

Life sciences study design

All studies must disclose on these points even when the disclosure is negative.

Sample size	For cryo-EM data collection, 2236 micrographs were collected from Quantifoil R 1.2/1.3 Cu 300 mesh grids for dataset 1. And 3358 micrographs were collected from GF 1.2/1.3 AU 300 mesh grids for dataset2. Cryo-EM data were obtained on a Titan Krios electron microscope operated at 300 kV using the super-resolution mode with a nominal magnification of 165 K.<br>For in vitro RNA cleavage gel assay, and fluorescence plate reader assay at least three independent biological replicates were performed to attain statistically firm results. For flow cytometry assay, 20'000 cells per sample and a minimum of 3 samples were analyzed to attain statistically firm results. No sample size predetermination was performed. The reported sample size was based on published works and was sufficient to obtain reproducible and reliable data for analysis.
Data exclusions	No data were excluded.
Replication	For in vitro RNA cleavage gel assay, fluorescence plate reader assay, and flow cytometry assay at least three independent biological replicates were performed. For the electrophoretic mobility shift assay, two independent biological replicates were performed. Experimental findings were reliably reproduced.
Randomization	Cells were randomly allocated into control and treatment groups, and samples used for flow cytometry assays were analyzed in random order.
Blinding	Blinding is not relevant to this study, all experiments were performed based on standardized protocols and readouts and are not influenced by the investigator.

Reporting for specific materials, systems and methods

We require information from authors about some types of materials, experimental systems and methods used in many studies. Here, indicate whether each material, system or method listed is relevant to your study. If you are not sure if a list item applies to your research, read the appropriate section before selecting a response.

Materials & experimental systems

n/a	Involved in the study	
☒	☐	Antibodies
☐	☒	Eukaryotic cell lines
☒	☐	Palaeontology and archaeology
☒	☐	Animals and other organisms
☒	☐	Clinical data
☒	☐	Dual use research of concern
☒	☐	Plants

Methods

n/a	Involved in the study	
☒	☐	ChIP-seq
☐	☒	Flow cytometry
☒	☐	MRI-based neuroimaging

Eukaryotic cell lines

Policy information about cell lines and Sex and Gender in Research

Cell line source(s)	HEK293T cells (ATCC CRL-3216)
Authentication	Order from American Type Culture Collection (ATCC). No additional authentication was performed by the authors of this study.
Mycoplasma contamination	All cell lines were tested negative for mycoplasma contamination.
Commonly misidentified lines (See ICLAC register)	No commonly misidentified cell lines were used.

Flow Cytometry

Plots

Confirm that:

☒ The axis labels state the marker and fluorochrome used (e.g. CD4-FITC).

☒ The axis scales are clearly visible. Include numbers along axes only for bottom left plot of group (a 'group' is an analysis of identical markers).

☒ All plots are contour plots with outliers or pseudocolor plots.

☒ A numerical value for number of cells or percentage (with statistics) is provided.

Methodology

Sample preparation	HEK293T cells (American Type Culture Collection, CRL-3216) were cultured in 10 cm treated, vented plates (Greiner Bio-One). The culturing media was Dulbecco's modified Eagle's Medium (DMEM) plus GlutaMAX (Gibco) which contained fetal bovine serum at 10% v/v (Gibco) and Penicillin-Streptomycin 1% v/v (Gibco). Cells were passaged between 80-90% confluency. The cell status used in all experiments was between Passage 1 to Passage 10. They were grown in an incubator set at 37 °C and 5% CO2. To prepare for an experiment, cells were seeded at a concentration of 20'0000 cells in 500 l of DMEM, with cell vitality above 95% and plate confluency around 90%. After seeding, cells were left at room temperature for 15 minutes to mitigate evaporation. After twelve hours, cells were transfected. For each well, 800 ng of plasmids were buffered to a final volume of 25 l in Opti-MEM I Reduced Serum Medium (OMEM) (Thermo Fisher Scientific). Along with the DNA, 2.5 g of Polyethyleneimine (PEI) (Thermo Fisher Scientific) was buffered to a final volume of 25 l in OMEM. The two were mixed and allowed to incubate at room temperature for 10 minutes before the total 50 l was transfected into each well.
Instrument	SONY SA3800
Software	Flowio v10.4.0
Cell population abundance	Cell vitality was assayed through Typhan Blue (Invitrogen) staining, placed on a Countess Cell Counting Chamber Slide (Invitrogen) and counted using a Countess II FL (Thermo Fisher Scientific), with the average of the two concentration and vitality measurements used. 100 % purity of cells of interest were used.
Gating strategy	The flow cytometry data was gated according to forward and side scatter to exclude debris and doublets.

☒ Tick this box to confirm that a figure exemplifying the gating strategy is provided in the Supplementary Information.
